# Bulletin of the Buffalo General Hospital

**Published:** 1912-10

**Authors:** 


### Bulletin of the Buffalo General Hospital

VOL. 2 OCTOBER, 1912. No. 2.

### Special Nursing.

The Hospital, on September 1st, returned to the system of nursing in use previous to the adoption of the twelve hour plan for special nursing. While the twelve hour system is still thought to be the ideal plan for nursing, it does not seem, at the present time, to meet the needs of the greatest number who desire the benefits of the Hospital. The Hospital desires that its benefits reach the the greatest number possible, and therefore has returned to the plan which it formerly was practicing.

---

### An Important Publication.

The Buffalo General Hospital is really a large institution, and is doing a tremendous amount of work. Because of its reputation and that of its staff, many cases of extreme rarity or interest find their way into its wards or private rooms. In this respect it is excelled by no institution of its size in the country. The institution has, in time past, partially failed in one important respect,—namely, in the publication of its valuable work to the professional public. Other institutions of much less importance have issued occasional reports, or are issuing annual Medical and Surgical Reports, which often contain case reports of great value. It will be welcome news to the friends of the Buffalo General Hospital, and to the hundreds of students who have studied within its walls, that the Medical Staff is now preparing its first Report of this character, which, when issued, will make a goodly sized volume of three hundred to five hundred pages, freely illustrated, and made up of reports of some of the remarkable cases of recent years, and of papers, by various members of the Staff, based upon experience gained within its limits.

The exact size of this volume will depend much upon the encouragement received from the Alumni and our professional friends generally. It would be most desirable that such a report should appear annually, although its preparation will entail no small labor, especially on the part of the committee entrusted by the Staff with its preparation. It is urged that those who would be willing to contribute either to its pages or to its financial support manifest their practical interest at the earliest possible date. It is intended that the book shall be as creditable to the institution as is the work which it is expected to record.

---

**Clinical Teaching.**

With the opening of the college year in our University formal clinical teaching has begun. So far as possible the work is arranged as follows:—Tuesday and Saturday are intended to be the principal public clinic days, during which operations are demonstrated. The work on Monday and Thursday is rather of the character of ward instruction, which, while even more important, is less spectacular. On Wednesday and Friday there is less of formal clinic work, but there are likely to be just as many operations or even more, especially those of a private character. Public clinics begin at half-past ten a. m., and continue until the work is finished, usually consuming at least two hours and often three. To these clinics all professional colleagues are cordially invited, as, in fact, they are to all surgical teaching in the Hospital, and to such operative work as is not necessarily of private character.

---

The apparatus for the production of anæsthesia by intratracheal insufflation, which has now been in service for a number of months, and to which reference was made in a previous issue, has given the greatest satisfaction, and is proving itself of the greatest practical value, particularly in operations about the chest, neck and head. When once in operation it is managed with the utmost ease, while the conduct of the anaesthesia is a matter of beautiful simplicity. The patient being relieved from all necessity for disquieting or troublesome respiration, the operator is thereby relieved from most of the anxiety which a conscientious surgeon feels at such a time; moreover, patients come out from under the influence of the anaesthetic, administered in this way, with scarcely any of the unpleasant after effects which seem unavoidable by ordinary methods.

---

In spite of additions and changes made in the Surgical Clinic the amount of surgical work done in the Hospital is now sufficient to make us feel, at times, uncomfortably crowded. It not infrequently happens that operations will be going on in four different rooms at the same time. This is stated simply to indicate the growth of the institution, and the fact that the adequate comforts of yesterday may become the crowded inconveniences of today, as is unavoidably the case in all growing institutions.

---

During the summer the Nurses' Home has been very thoroughly renovated, through the generosity of Mrs. Hamlin and Mrs. Pardee. Hard wood floors have been laid, walls painted, and the building made bright and attractive throughout. Its housing capacity will be well tested this winter, for the largest class of probationers yet received—twenty-four in number—entered in September.

---

Dr. Ross gave a paper on "The Use of Salvarsan in Hospitals" at the meeting of the American Hospital Association at Detroit in September.

---

The maternity service has now fourteen free beds at its disposal. No preliminary arrangements are necessary for admission to these beds, but patients in need may apply in person, or the ambulance will be sent for them. Many critical and difficult cases are brought in on "hurry calls." Every patient receives skillful and conscientious attention.

---

**The Free Bed.**

Thirty-eight years ago the first free bed was established at the General Hospital, through the gift of five thousand dollars by Mrs. Lavinia H. Austin. This notable benefaction was followed in due time by two others, of like amount, from Mr. James N. Matthews and Mr. Arthur McArthur, respectively, and in 1884 was founded the first free bed for children, through a bequest of three thousand dollars from Mrs. J. B. Skinner. This was a peculiarly appropriate gift. Mrs. Skinner had been an especially valuable friend of the Hospital, and was for many years president of the Ladies' Board, which had first urged the establishment of a children's ward. Soon after this the Sunday Schools of Trinity and the First Church gave each three thousand dollars to endow a bed in the children's ward, and so appealing is this form of endowment, in general, that the General Hospital has now eighteen such permanent foundations. This does not include the maternity service, which is supported by yearly gifts. In 1905 the uniform endowment of five thousand dollars was established for the free bed.

The devoted workers for the hospital, in its early days, would have considered such provision in this direction as the institution now enjoys lavish indeed—but what does this equipment mean today, in power to meet the needs which it is supposed to serve? In 1874, when Mrs. Austin made that important initial gift, five thousand dollars yielded $350.00 a year; the weekly cost per patient was $7.15; the bed was therefore self-sustaining for forty-eight weeks and a fraction—practically the entire year. In 1884, when the first three thousand dollar bed was established, the income from it was at least $180.00 a year, and the weekly cost per patient was $7.00. Those were the happy housekeeping days when eggs were unconsidered trifles, and milk cost the hospital one dollar a year for each patient. Today, five thousand dollars yields about $240.00, and the weekly cost per patient has risen to $11.65. The bed can be "warm," without cost to the hospital, for less than half the year.

This shrinkage is due not only to the two causes already indicated, sadly familiar to the outside world as well, but to the revolution in hospital procedure in the last twenty-five years. When, for instance, the surgeons put on rubber gloves, they reduced the mortality rate, but increased the annual expenses by about $1,000—the cost of living, as it were.

And yet the free bed is now at its highest social value. The peculiar aid which it renders to the worthy poor we all recognize; they never needed its assistance more; while in the new struggle with destructive forces in which society has engaged it is a potent instrument. Through it many a stream of disease and vice is checked, when by no other means could the delinquent or the victim be reached. Every hospital could report tangles of vice, degeneracy and criminal irresponsibility, where the free bed cleared a little space in the wretched welter, and by so much served the public welfare.

---

**That Hospital Deficit.**

The International Hospital Record stated in a recent issue that the current expenses of forty-five hospitals in New York City have this year exceeded their annual income "from invested funds, paying patients, and city appropriations by about $1,500,000." The Record goes on to say that "the wonder is, with operating expenses increasing so heavily, and also the living expenses of the general public, that many hospitals, not alone in New York City, but elsewhere, are not compelled either largely to limit their charity, or to close their doors."

It is surely the business of a hospital to live within its income, or at least within its reasonable expectation of income; yet few persons realize what conditions a hospital has to meet. Let us cite a few examples.

In one of our wards is a woman who entered as a county case a year and a half ago. As the time approached for her discharge the social workers did all in their power to induce her relatives to give her a home, for she was helpless for life. Her disease had necessitated the amputation of both legs. At last one of her sisters consented to receive her, and the ambulance took her off. But the interval, though brief, had been fatal. When the sister's home was reached, they found that she had locked the door and beat a thrifty retreat. The attendants, having marched upstairs with their patient, had no alternative but to follow a famous historic example and march down again. At this writing, the patient is still in the hospital, entirely a charge upon its resources, the county having refused to carry her any longer.

Not long ago a man was brought in from the country, badly burned. He had neither friends nor money, and had been in this country so short a time that he had no claim on the public funds. He was cared for until he was on his feet again. A similar case was of a woman who had been in this country but a short time, and was brought to the hospital for a serious operation. She, too, was entirely without resources, and was of a kind to feel her friendlessness keenly. During her convalescence work was found for her which was much better than that which she had had before, and she left the Hospital declaring that the misfortune which took her there was the luckiest thing that ever happened to her.

A young woman suffering from tubercular spine was brought to the hospital. She, too, had been here too short a time to receive public assistance. She was taken care of for almost four months.

In none of these cases could the Hospital, in humanity, have done less. Yet for the last three patients it received no compensation whatsoever; for the crippled woman nothing was received for some months, while for the remainder of the year and a half the county paid for her $5.50 a week. The weekly cost per patient this year, it will be remembered, is $11.65. Here we see, in large measure, the stuff of which hospital deficits are made. We see also that they are involved with one of the urgent public questions of the day.

